# Transcriptomes analysis reveals novel insight into the molecular mechanisms of somatic embryogenesis in *Hevea brasiliensis*

**DOI:** 10.1186/s12864-021-07501-9

**Published:** 2021-03-12

**Authors:** Ying Wang, Hui-Liang Li, Yong-Kai Zhou, Dong Guo, Jia-Hong Zhu, Shi-Qing Peng

**Affiliations:** 1 Key Laboratory of Biology and Genetic Resources of Tropical Crops, Ministry of Agriculture, Institute of Tropical Bioscience and Biotechnology, Chinese Academy of Tropical Agricultural Sciences, No.4 Xueyuan Road, Haikou, 571101 China; 2 School of Life and Pharmaceutical Sciences, Hainan University, Haikou, 570228 China; 3 Hainan Academy of Tropical Agricultural Resource, CATAS, Haikou, 571101 China

**Keywords:** *Hevea brasiliensis*, Somatic embryogenesis, RNA-seq, Hormone signal, Transcription factor, Histone modification

## Abstract

**Background:**

Somatic embryogenesis (SE) is a promising technology for plant vegetative propagation, which has an important role in tree breeding. Though rubber tree (*Hevea brasiliensis* Muell. Arg.) SE has been founded, few late SE-related genes have been identified and the molecular regulation mechanisms of late SE are still not well understood.

**Results:**

In this study, the transcriptomes of embryogenic callus (EC), primary embryo (PE), cotyledonary embryo (CE), abnormal embryo (AE), mature cotyledonary embryo (MCE) and withered abnormal embryo (WAE) were analyzed. A total of 887,852,416 clean reads were generated, 85.92% of them were mapped to the rubber tree genome. The de novo assembly generated 36,937 unigenes. The differentially expressed genes (DEGs) were identified in the pairwise comparisons of CE vs. AE and MCE vs. WAE, respectively. The specific common DEGs were mainly involved in the phytohormones signaling pathway, biosynthesis of phenylpropanoid and starch and sucrose metabolism. Among them, hormone signal transduction related genes were significantly enriched, especially the auxin signaling factors (*AUX-like1*, *GH3.1*, *SAUR32-like*, *IAA9-like*, *IAA14-like*, *IAA27-like*, *IAA28-like* and *ARF5-like*). The transcription factors including *WRKY40*, *WRKY70*, *MYBS3-like*, *MYB1R1*-*like*, *AIL6* and *bHLH93-like* were characterized as molecular markers for rubber tree late SE. *CML13*, *CML36*, *CAM-7*, *SERK1* and *LEAD-29-like* were also related to rubber tree late SE. In addition, histone modification had crucial roles during rubber tree late SE.

**Conclusions:**

This study provides important information to elucidate the molecular regulation during rubber tree late SE.

**Supplementary Information:**

The online version contains supplementary material available at 10.1186/s12864-021-07501-9.

## Background

Rubber tree (*Hevea brasiliensis* Muell. Arg.), a tropical rubber-producing tree within the *Euphorbiaceae* family which is native to the great Amazonian basin of South America, is now widely cultivated to produce natural rubber in Southeast Asia [1]. Rubber tree is a perennial cross-pollination tree with a long juvenile period, which makes low efficiency of hybrid breeding [2]. Rubber tree is still propagated mostly by grafting, although the interaction between scion and rootstock of the grafted tree affects the growth, and natural rubber yield [3, 4].

Somatic embryogenesis (SE) is a promising and rapid vegetative propagation technique for plant regeneration. Plant regeneration via SE process in rubber tree had been established using different kinds of explants including immature anthers, internal integuments of immature fruits, inflorescence, as well as root [5–8]. The regenerated plants have juvenile characters and their own roots, which are called self-rooted juvenile clones (SRJCs). Compared with donor clones, SRJCs is superior in growth, rubber yield and stress resistance [9–11], which is a promising new rubber tree planting material in the future. There are two pathways (indirect primary SE, direct primary SE) to obtain primary somatic embryos [11]. Secondary SE allows to produce an unlimited number of secondary somatic embryos in a cyclic routine [10]. At present, the SE process is limited by irregular germination of the somatic embryos and low efficiency of plantlet recovery from somatic embryos [11], only a limited number of rubber tree genotypes can obtain regeneration plant [11–15].

To study the molecular regulation mechanisms of plant SE, the analyses of transcriptomes were carried out to identify SE related genes by RNA-seq in plant species, including herbaceous plants such as *Arabidopsis* [16], *Gossypium hirsutum* [17], maize [18], strawberry [19], rice [20], and woody plants such as Norway spruce [21], coconut plam [22], Brazilian pine [23], camphor tree [24], papaya [25], *Dimocarpus longan* [26] and so on. These studies demonstrated regulation mechanisms of SE at a molecular level, and several potential key genes were identified, such as genes encoding late embryogenesis abundant (LEA) protein [25], somatic embryogenesis receptor-like kinase (SERK) [27, 28], Leafy Cotyledon [28, 29], AGAMOUS-like 15 [30, 31], BBM (BABY BOOM) [28, 32], WUSCHEL [33, 34], and WUSCHEL homeobox 2 [28, 35].

SE of rubber tree can only be obtained for a limited number of genotypes [12–14]. Few studies have reported the molecular regulation mechanism of rubber tree SE. For example, Charbit et al. found that five cDNAs were differentially expressed in the embryogenic regenerating line could be enable an early diagnosis of friable rubber tree callus embryogenic potential, but the functions of these cDNAs haven’t been identified [12]. Li et al. [36] found that three MADS-box genes (genes encode transcription factors that promote SE in many plant species [37–39]), were differentially expressed during rubber tree early embryogenesis , suggesting MADS-box genes involved rubber tree SE. Piyatraku et al. reported that 11 AP2/ERF genes might act as expression markers linked to the different stages of the somatic embryogenesis process in rubber tree [14]. Some studies have also shown that AP2/ERF genes play important roles in somatic embryogenesis as these genes involved in SE regulation [40–42]. However, the molecular regulation mechanisms of the late stage of rubber tree SE are still not well understood. To clarify whether the regenerate competence of different embryos depend on the genes during late SE, we investigated the expression profiling using RNA-seq technology. This study will offer valuable information for the molecular regulation mechanisms of rubber tree late SE.

## Results

### Induction of somatic embryogenesis

**Fig. 1:**
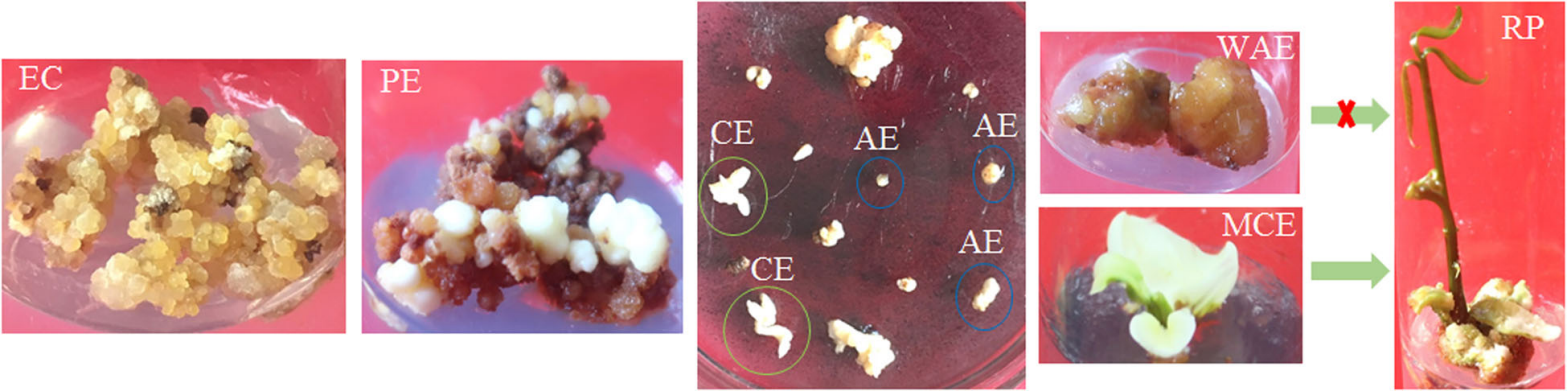
The cultures during *H. brasiliensis* SE. EC: embryogenic callus; PE: primary embryo; CE: cotyledonary embryo; MCE: mature cotyledonary embryo; AE: abnormal embryo; WAE: withered abnormal embryo

The procedure of somatic embryogenesis and regeneration in *H. brasiliensis* was established (Fig. 1) as described previously [5]. The immature anthers were cultured in solid Murashige and Skoog (MS) medium supplemented with 2, 4-dichlorophenoxyacetic acid (2, 4 -D), kinetin (KT) and naphthylacetic acid (NAA) for 50 days. At the end of the period, the embryogenic calluses (ECs) were obtained. ECs were placed in the MS medium containing indole-3-acetic acid (IAA) and gibberellic acid (GA_3_) for embryo induction. After 40 days, primary embryos (PEs) were collected. The PEs were transferred to MS medium containing 6-benzyl aminopurine (6-BA) and AgNO_3_ for growing. After 40 days, two different embryos based on their phenotype (cotyledonary embryo (CE), abnormal embryo (AE)) were observed in the culture medium.. We observed a significant difference between CEs and AEs in phenotype. The CEs and AEs were placed on half-strength MS medium containing IAA and BA. The CEs turned stronger into the mature cotyledonary embryo (MCE) 20 days later, whereas the AEs turned brown and grown up into withered abnormal embryo (WAE). After 30 days, the MCEs grew into complete seedlings, whereas the WAEs turned black and died. Based on the above phenotypic observation, six different samples during SE were selected for further study.

### Transcriptome analysis of rubber tree SE

High-throughput sequencing generated 915,535,874 raw reads in EC, PE, CE, AE, MCE and WAE samples. A total of 887,852,416 clean reads were retained by filtering the reads with adaptor sequences and ambiguous “N” base. The percentage of quality score above 30 (Q30) was 97.92% and the GC percentage was 43% (Table 1). On average, 85.92% of the clean reads were mapped to *H. brasiliensis* genome.

**Table 1:** Pre-processing statistics and quality control statistics

Sample	Raw Reads	Clean Reads	Raw Bases (Gb)	Clean Bases (Gb)	Effective Rate (%)	Q30 content (%)
EC-1	5.2E+ 07	50,059,934	7.86	7.56	96.21	94.81
EC-2	5.1E+ 07	49,524,648	7.73	7.48	96.73	94.81
EC-3	5.1E+ 07	49,118,950	7.68	7.42	96.61	94.78
PE-1	5E+ 07	48,319,634	7.53	7.25	96.29	97.01
PE-2	5.1E+ 07	49,061,282	7.64	7.36	96.33	96.86
PE-3	5.1E+ 07	48,891,852	7.6	7.33	96.46	96.9
CE-1	5.1E+ 07	49,805,096	7.73	7.52	97.32	94.74
CE-2	5.2E+ 07	50,906,314	7.88	7.69	97.56	94.91
CE-3	5.1E+ 07	50,054,842	7.76	7.56	97.4	94.84
MCE-1	5.1E+ 07	49,771,578	7.7	7.47	96.96	95.89
MCE-2	5E+ 07	48,654,566	7.54	7.3	96.85	94.92
MCE-3	5E+ 07	48,974,062	7.52	7.35	97.72	95.62
AE-1	5E+ 07	48,881,230	7.56	7.33	97.05	96.81
AE-2	5.1E+ 07	48,970,492	7.6	7.35	96.7	96.75
AE-3	5.1E+ 07	48,844,568	7.59	7.33	96.52	96.88
WAE-1	5.1E+ 07	49,843,978	7.71	7.53	97.67	94.71
WAE-2	5E+ 07	49,076,246	7.6	7.41	97.49	94.65
WAE-3	5E+ 07	49,093,144	7.59	7.41	97.71	94.72

**Fig. 2:**
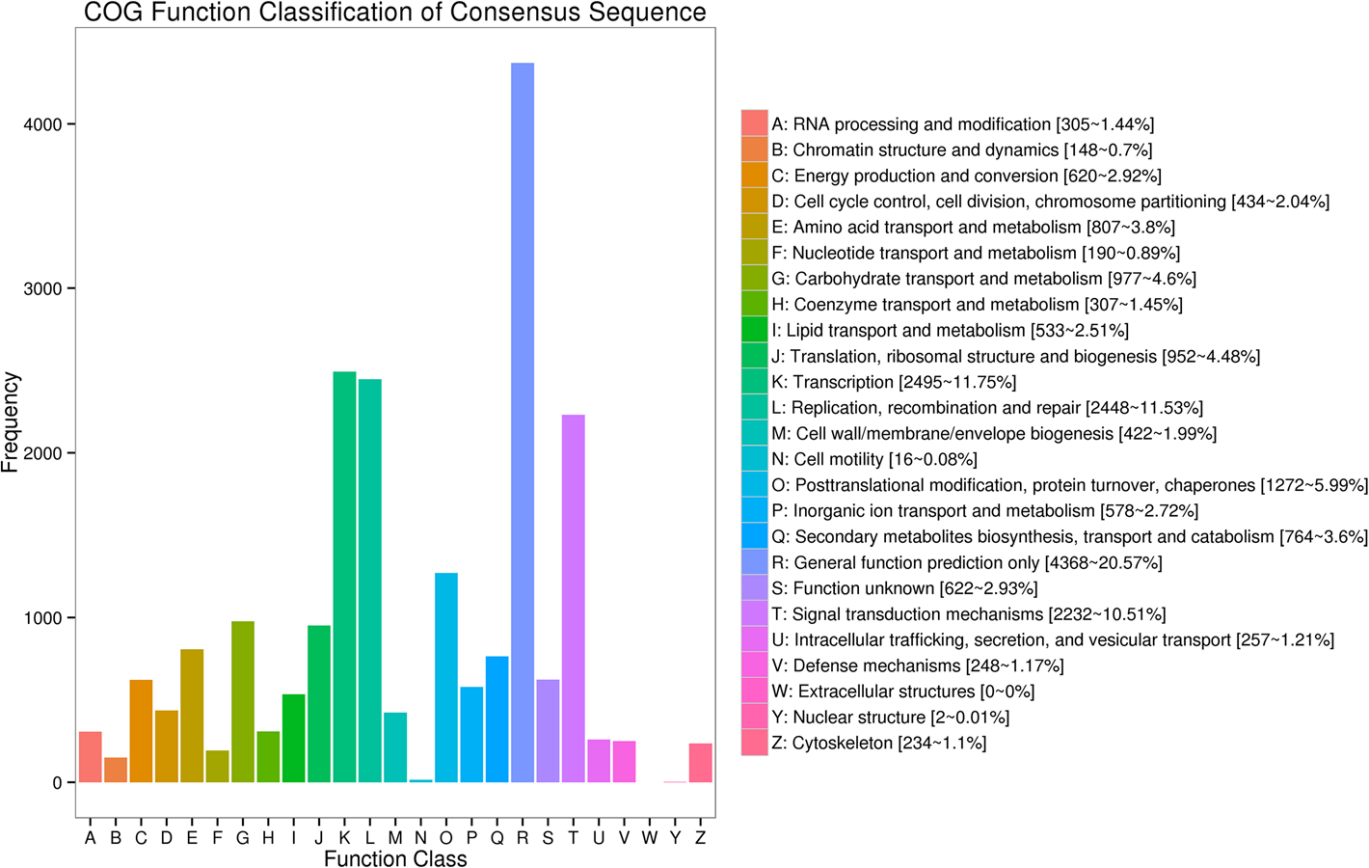
The COG assignments of assembled unigenes. Out of 36,937 de novo assembled unigenes, 13,421 were assigned to 25 COG categories GO annotation of assembled unigenes by Blast2GO during *H. brasiliensis* SE

All unigenes were annotated by the blast search against the public databases using BLASTx (E-value–5 ≤ 10). All 36,937 unigenes were annotated in 4 databases involved in the Clusters of Orthologous Groups of proteins (COG) database, the Gene Ontology (GO) database, the clusters of euKaryotic Orthologous Groups (KOG) database and the Evolutionary Genealogy of Genes: Non-supervised Orthologous Groups (eggNOG) database (Table 2). According to the COG functional classification, the 13,421 unigenes were categorized into 25 COG categories. The four most highly represented COG categories were “general function prediction only” (20.57%), “transcription” (11.75%), “replication, recombination and repair” (11.53%) and “signal transduction mechanisms” (10.51%)(Fig. 2). In addition, 19,619, 20,954 and 36,362 unigenes were successfully annotated in GO, KOG, eggNOG, respectively (Fig. S1, S2, S3).

**Table 2:** The number and distribution of unigenes annotated in the databases

Database	Annotated Number	300 < =length < 1000	length > =1000
COG	13,421	4142	9153
GO	19,619	5980	13,639
KOG	20,954	7547	13,097
eggNOG	36,362	14,578	21,038
All	36,937	14,983	21,176

### Global analysis of gene expression during rubber tree

**Fig. 3:**
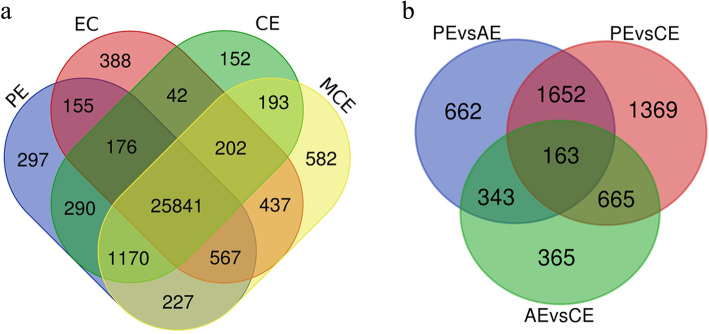
Statistical analysis of the DEGs during SE stages. **a** The venn diagram of expressed genes in four developmental stages. **b** The venn diagram of expressed genes in PE vs. AE, PE vs. CE and CE vs. AE. EC: embryogenic callus; PE: primary embryo; CE: cotyledonary embryo; AE: abnormal embryo; MCE: mature cotyledonary embryo; WAE: withered abnormal embryo

A Venn diagram was created to find the overlapped genes in the four different developmental stages of *H. brasiliensis* SE (Fig. 3a). A total of 25,841 genes overlapped in the four stages. Among them, 155 genes overlapped between EC and PE; 290 genes overlapped between PE and CE; 193 genes overlapped between CE and MCE. A total of 388, 297, 152 and 582 genes were uniquely expressed in EC, PE, CE and MCE respectively. Another Venn diagram was also created to find the overlapped genes in the comparisons of PE, AE and CE of *H. brasiliensis* SE (Fig. 3b). As shown in Fig. 3b, 662 genes were exclusive to PE vs. AE. 1369 genes were exclusive to PE vs. CE. Moreover, 365 genes were found in AE vs. CE. To evaluate the differences of molecular response among all samples, the expression level of the unigenes was calculated by the expected number *of* Fragments Per Kilobase of transcript sequence per Million base pairs sequenced (FPKM). The top 20 expressed genes from EC, PE, CE and MCE libraries were shown in Table 3. Some of them including *glutathione S-transferase (GST)*, *lipid-transfer protein (LTP)*, *peroxidase (POD)*, *indole-3-acetic acid-amido synthetase GH3.1*, *ADP-ribosylation factor*, *catalase isozyme*, and *polyubiquitin,* were highly expressed in four stages.

**Table 3:** The top 20 expressed genes in EC, PE, CE and MCE library

		No	Gene-ID	Database-ID	FPKM-EC	Description
**EC library**		1	gene10318	XM_021818345.1	3266.37	metallothionein-like protein type 2
		2	gene23077	XM_021779607.1	2803.41	probable indole-3-acetic acid-amido synthetase GH3.1
		3	gene24550	XM_021781891.1	2587.98	peptidyl-prolyl cis-trans isomerase-like
		4	gene37167	XM_021801099.1	5591.156667	pathogenesis-related protein PR-4-like
		5	gene41379	XM_021807510.1	3354.676667	metallothionein-like protein type 3
		6	gene41538	XM_021807736.1	1336.78	peroxidase 12-like
		7	gene42156	XM_021808475.1	3150.052519	L-ascorbate peroxidase, cytosolic-like
		8	gene548	XM_021811448.1	1719.636667	thioredoxin H-type-like
		9	gene11066	XM_021819455.1	1216.968149	catalase isozyme 2-like
		10	gene1185	XM_021821602.1	6138.533333	metallothionein-like protein type 2
		11	gene15002	XM_021825368.1	2578.13	glucan endo-1,3-beta-glucosidase, basic isoform-like
		12	gene18326	XM_021830411.1	2459.693333	endochitinase EP3-like
		13	gene19193	XM_021831939.1	1835.053335	glutathione S-transferase F9-like
		14	gene33311	XM_021795239.1	1328.469977	pathogenesis-related protein PR-4-like
		15	gene3644	XM_021801975.1	1588.716667	thaumatin-like protein 1b
		16	gene41464	XM_021807622.1	2882.38	endochitinase EP3-like
		17	gene5134	XM_021810359.1	2157.947846	catalase isozyme 2
		18	gene12558	XM_021821637.1	1745.217667	cysteine synthase
		19	gene21974	XM_021836019.1	1238.357898	40S ribosomal protein S25–3-like
		20	gene24408	XM_021781690.1	1518.806667	polyubiquitin
**PE library**		1	gene17338	XM_021828886.1	448.8675164	ADP-ribosylation factor
		2	gene24550	XM_021781891.1	1090.893333	peptidyl-prolyl cis-trans isomerase-like
		3	gene25944	XM_021784022.1	517.586	polyubiquitin
		4	gene37168	XM_021801110.1	1051.049333	pathogenesis-related protein PR-4-like
		5	gene37235	XM_021801218.1	424.1643333	probable glutathione S-transferase
		6	gene5278	XM_021810573.1	700.6816667	probable aquaporin TIP3–2
		7	gene548	XM_021811448.1	651.8526667	thioredoxin H-type-like
		8	gene1185	XM_021821602.1	691.8516667	metallothionein-like protein type 2
		9	gene17500	XM_021829184.1	651.8516667	uncharacterized
		10	gene19193	XM_021831939.1	444.690335	glutathione S-transferase F9-like
		11	gene19425	XM_021832135.1	4129.713333	non-specific lipid-transfer protein 1-like
		12	gene22222	XM_021836400.1	475.7673333	histone H2B
		13	gene23940	XM_021780963.1	563.119	osmotin-like protein
		14	gene37576	XM_021801775.1	574.5693333	thaumatin-like protein
		15	gene12558	XM_021821637.1	419.428	cysteine synthase
		16	gene35575	XM_021798790.1	464.6649333	copper transport protein ATX1-like
		17	gene30702	XM_021791318.1	1738.72	peroxidase 42-like
		18	gene23545	XM_021780391.1	2407.276667	peroxidase 42-like
		19	gene33942	XM_021796208.1	577.7063333	peptidyl-prolyl cis-trans isomerase 1
		20	gene24408	XM_021781690.1	472.838	polyubiquitin
**CE library**		1	gene17338	XM_021828886.1	1145.143911	ADP-ribosylation factor
		2	gene18178	XM_021830179.1	943.5483996	protein translation factor SUI1 homolog 2-like
		3	gene25944	XM_021784022.1	1762.396667	polyubiquitin
		4	gene37168	XM_021801110.1	9026.456667	pathogenesis-related protein PR-4-like
		5	gene37235	XM_021801218.1	2538.033333	probable glutathione S-transferase
		6	gene5278	XM_021810573.1	1959.873667	probable aquaporin TIP3–2
		7	gene5809	XM_021811329.1	1199.044333	metallothionein-like protein type 2
		8	gene7973	XM_021814772.1	1108.26	glutaredoxin
		9	gene9140	XM_021816591.1	4259.97	metallothionein-like protein type 2
		10	gene17500	XM_021829184.1	995.952	uncharacterized
		11	gene19425	XM_021832135.1	3340.51	non-specific lipid-transfer protein 1-like
		12	gene20309	XM_021833577.1	1361.93341	ubiquitin-conjugating enzyme E2 28
		13	gene12558	XM_021821637.1	1265.565333	cysteine synthase
		14	gene25797	XM_021783808.1	1133.149667	L-ascorbate peroxidase, cytosolic
		15	gene30702	XM_021791318.1	2923.166667	peroxidase 42-like
		16	gene23545	XM_021780391.1	3234.236667	peroxidase 42-like
		17	gene24345	XM_021781508.1	1190.793333	translationally-controlled tumor protein homolog
		18	gene36607	XM_021800241.1	1155.013333	aquaporin TIP1–1-like
		19	gene41316	XM_021807427.1	1017.603343	aquaporin PIP1–3-like
		20	gene31451	XM_021792523.1	1867.496667	probable aquaporin PIP1–2
**MCE library**		1	gene17338	XM_021828886.1	1126.976	ADP-ribosylation factor
		2	gene18178	XM_021830179.1	1004.106	protein translation factor SUI1 homolog 2-like
		3	gene25944	XM_021784022.1	2101.800	polyubiquitin
		4	gene33318	XM_021795235.1	1202.313	pro-hevein
		5	gene37168	XM_021801110.1	18,664.897	pathogenesis-related protein PR-4-like
		6	gene39161	XM_021804156.1	918.860	2-methylbutanal oxime monooxygenase
		7	gene41379	XM_021807510.1	864.198	metallothionein-like protein type 3
		8	gene41597	XM_021807803.1	791.620	elicitor-responsive protein 3-like
		9	gene42156	XM_021808475.1	2095.956	L-ascorbate peroxidase, cytosolic-like
		10	gene548	XM_021811448.1	831.757	thioredoxin H-type-like
		11	gene9140	XM_021816591.1	5217.597	metallothionein-like protein type 2
		12	gene11066	XM_021819455.1	1380.437	catalase isozyme 2-like
		13	gene1185	XM_021821602.1	1598.003	metallothionein-like protein type 2
		14	gene19425	XM_021832135.1	2686.840	non-specific lipid-transfer protein 1-like
		15	gene20309	XM_021833577.1	1215.717	ubiquitin-conjugating enzyme E2 28
		16	gene5134	XM_021810359.1	1842.001	catalase isozyme 2
		17	gene19423	XM_021832115.1	1306.773	non-specific lipid-transfer protein 1-like
		18	gene23545	XM_021780391.1	1473.353	peroxidase 42-like
		19	gene24345	XM_021781508.1	1151.487	translationally-controlled tumor protein homolog
		20	gene31451	XM_021792523.1	821.413	probable aquaporin PIP1–2

**Fig. 4:**
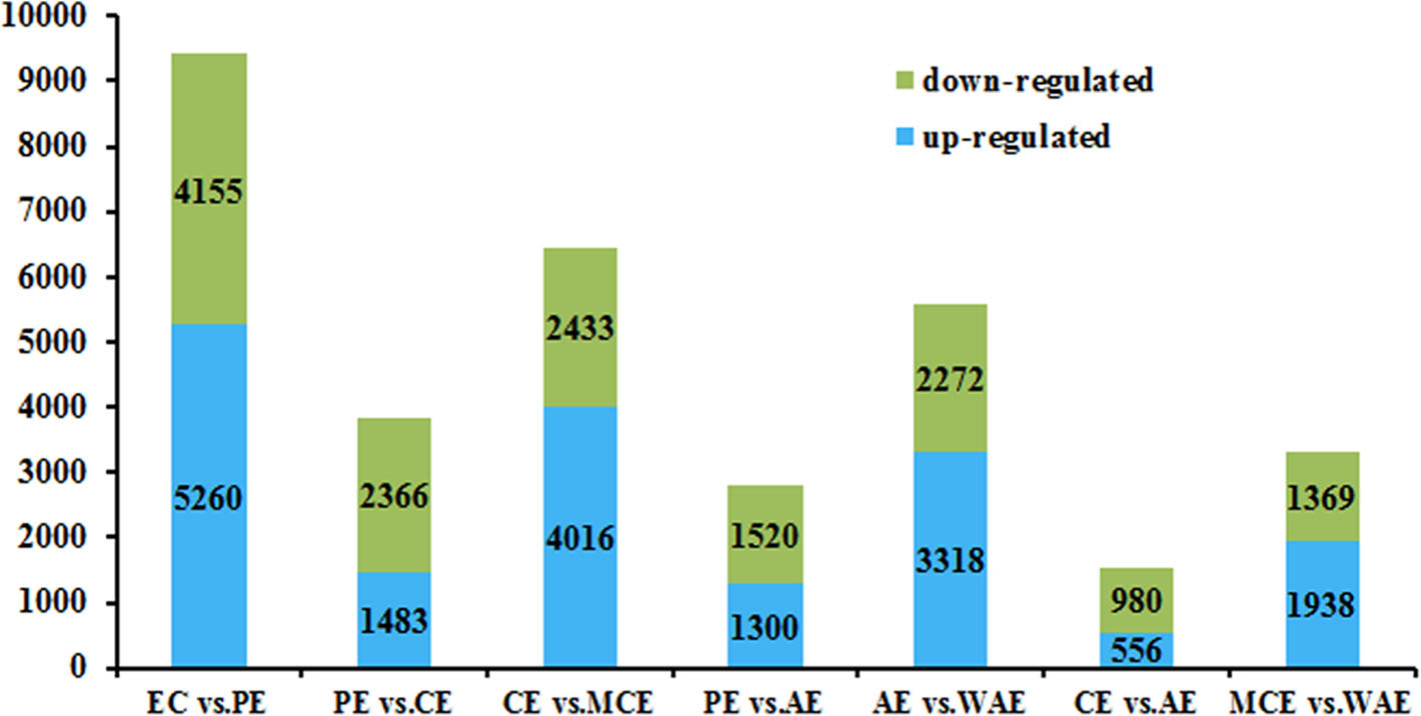
The number of up- or down-regulated DEGs in EC vs. PE, PE vs. CE, CE vs. MCE, PE vs. AE, AE vs. WAE, CE vs. AE, MCE vs. WAE. EC: embryogenic callus; PE: primary embryo; CE: cotyledonary embryo; AE: abnormal embryo; MCE: mature cotyledonary embryo; WAE: withered abnormal embryo

In order to reveal the potential key factors and deeply understand the regulatory network of SE, the unigenes of each library of *H. brasiliensis* SE were compared under the condition of − 1.0 ≥ Log_2_ [Fold Change (FC)] ≥ 1.0 and False Discovery Rate (FDR) < 0.01. A total of 9415 DEGs were obtained in EC vs. PE, PE had 5260 up-regulated and 4155 down-regulated genes. In PE vs. CE, CE had 1483 genes up-regulated and 2366 genes down-regulated. In CE vs. MCE, 6449 DEGs were obtained, of which 4016 DEGs were up-regulated, whereas 2433 DEGs were down-regulated. The 2820 DEGs were found in PE vs. AE with 1300 up-regulated and 1520 down-regulated DEGs. In AE vs. WAE, 5590 DEGs were obtained, of which 3318 DEGs were up-regulated, whereas 2272 DEGs were down-regulated. In AE vs. CE, 1536 DEGs were found with 556 up-regulated and 980 down-regulated DEGs. The 3307 DEGs were found between WAE vs. MCE with 1938 up-regulated and 1369 down-regulated DEGs (Fig. 4).

### GO analysis of DEGs

**Fig. 5:**
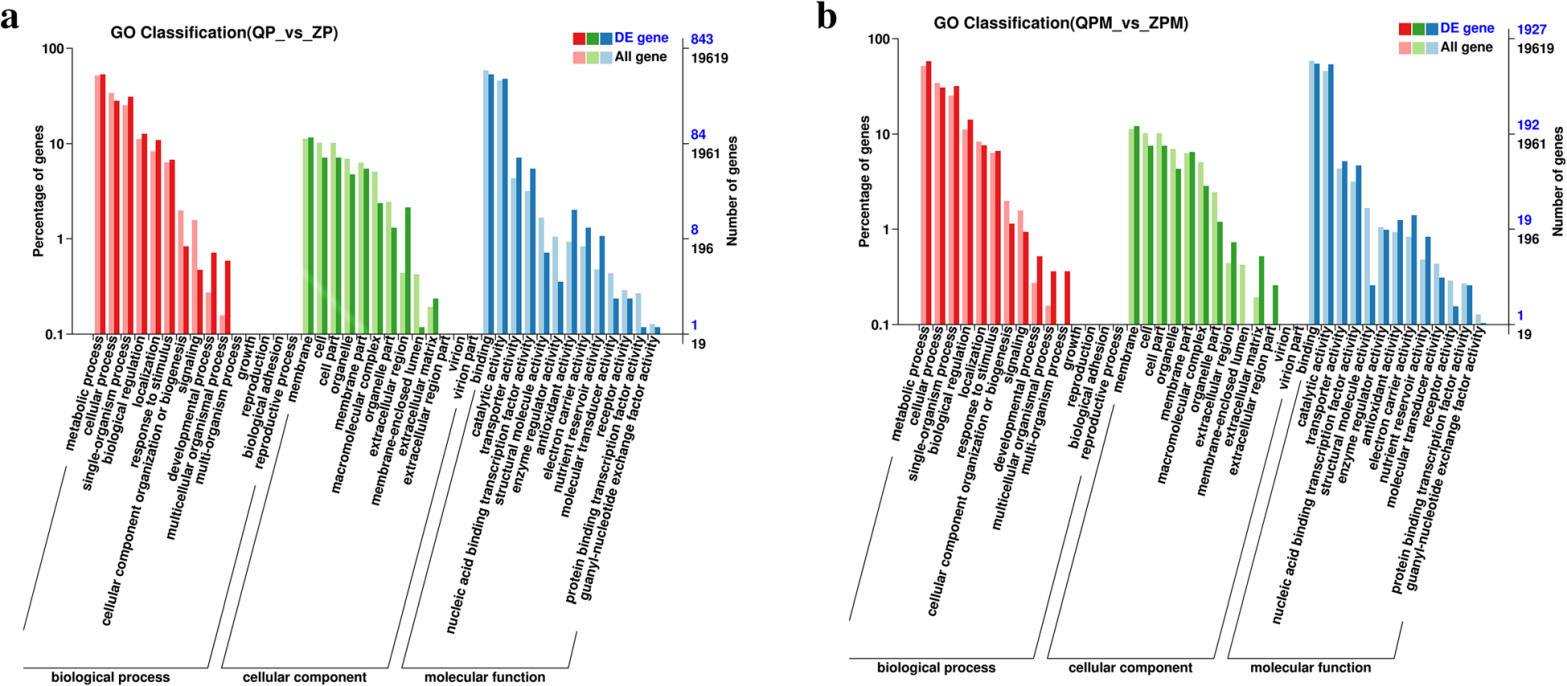
Molecular functions and biological processes of DEGs in CE vs. AE (**a**) and MCE vs. WAE (**b**) based on gene ontology categories. CE: cotyledonary embryo; AE: abnormal embryo; MCE: mature cotyledonary embryo; WAE: withered abnormal embryo

To further demonstrate the unigenes functions, GO assignments were carried out using the Blast2GO program. In AE vs. CE, 843 DEGs were classified into three major categories: biological processes (BP), cellular components (CC) and molecular function (MF). A total of 41 GO subcategories were enriched over three major functional categories. The main subcategories are shown in Fig. 5a. The six major subcategories of BP were metabolic process, cellular process, single-organism process, biological regulation, localization and response to stimulus. The five major subcategories of CC were membrane, cell, cell part, organelle and membrane part. The four major subcategories of MF were binding, catalytic activity, transporter activity and nucleic acid binding transcription factor activity. In WAE vs. MCE, 1927 DEGs were also classified into BP, CC and MF and subcategorized in 41 GO (Fig. 5b). The major subcategories of three categories were consistent with the result in AE vs. CE.

### Kyoto encyclopedia of genes and genomes (KEGG) pathway of DEGs

**Fig. 6:**
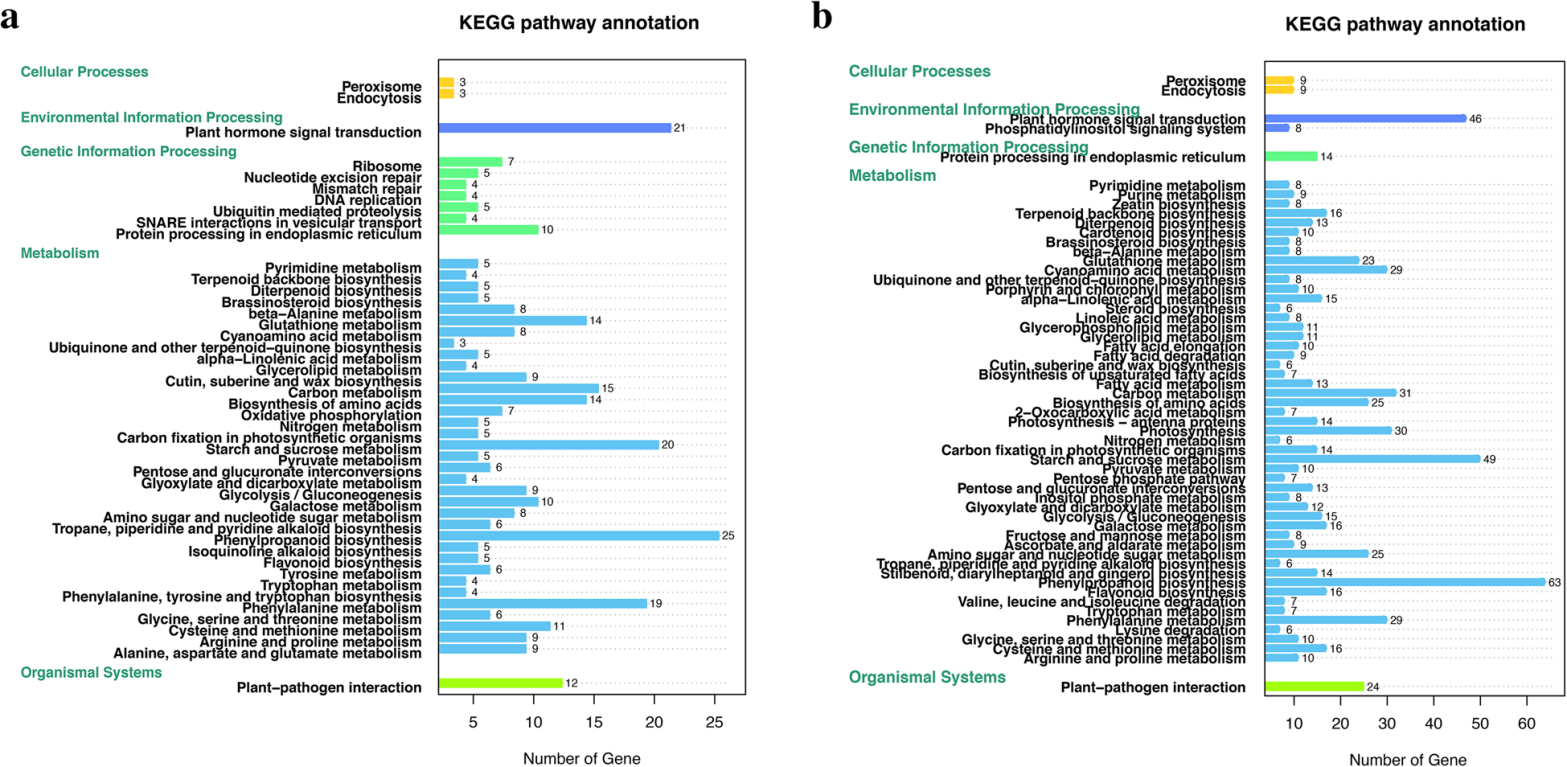
KEGG annotation of DEGs in CE vs. AE (**a**) and MCE vs. WAE (**b**) based on gene ontology categories. CE: cotyledonary embryo; AE: abnormal embryo; MCE: mature cotyledonary embryo; WAE: withered abnormal embryo

There were 376 DEGs in AE vs. CE, which were assigned to 46 KEGG pathways (Fig. 6a). The most representative pathways were phenylpropanoid biosynthesis (25 unigenes), plant hormone signal transduction (21 unigenes), starch and sucrose metabolism (20 unigenes), phenylalanine metabolism (19 unigenes), carbon metabolism (15 unigenes), biosynthesis of amino acid (14 unigenes) and glutathione metabolism (14 unigenes).

In WAE vs. MCE, the 771 DEGs were assigned to 57 KEGG pathways (Fig. 6b). The most represented pathways were phenylpropanoid biosynthesis (63 unigenes), starch and sucrose metabolism (49 unigenes), plant hormone signal transduction (46 unigenes), carbon metabolism (31 unigenes), photosynthesis (30 unigenes), phenylalanine metabolism (29 unigenes) and cyanoamino acid metabolism (29 unigenes). The results indicated that phenylpropanoid biosynthesis, phytohormones signaling pathway, and sucrose and starch metabolism played importance roles during *H. brasiliensis* late SE*.*

### Differential expression of hormone signal transduction related genes between CE and AE

**Fig. 7:**
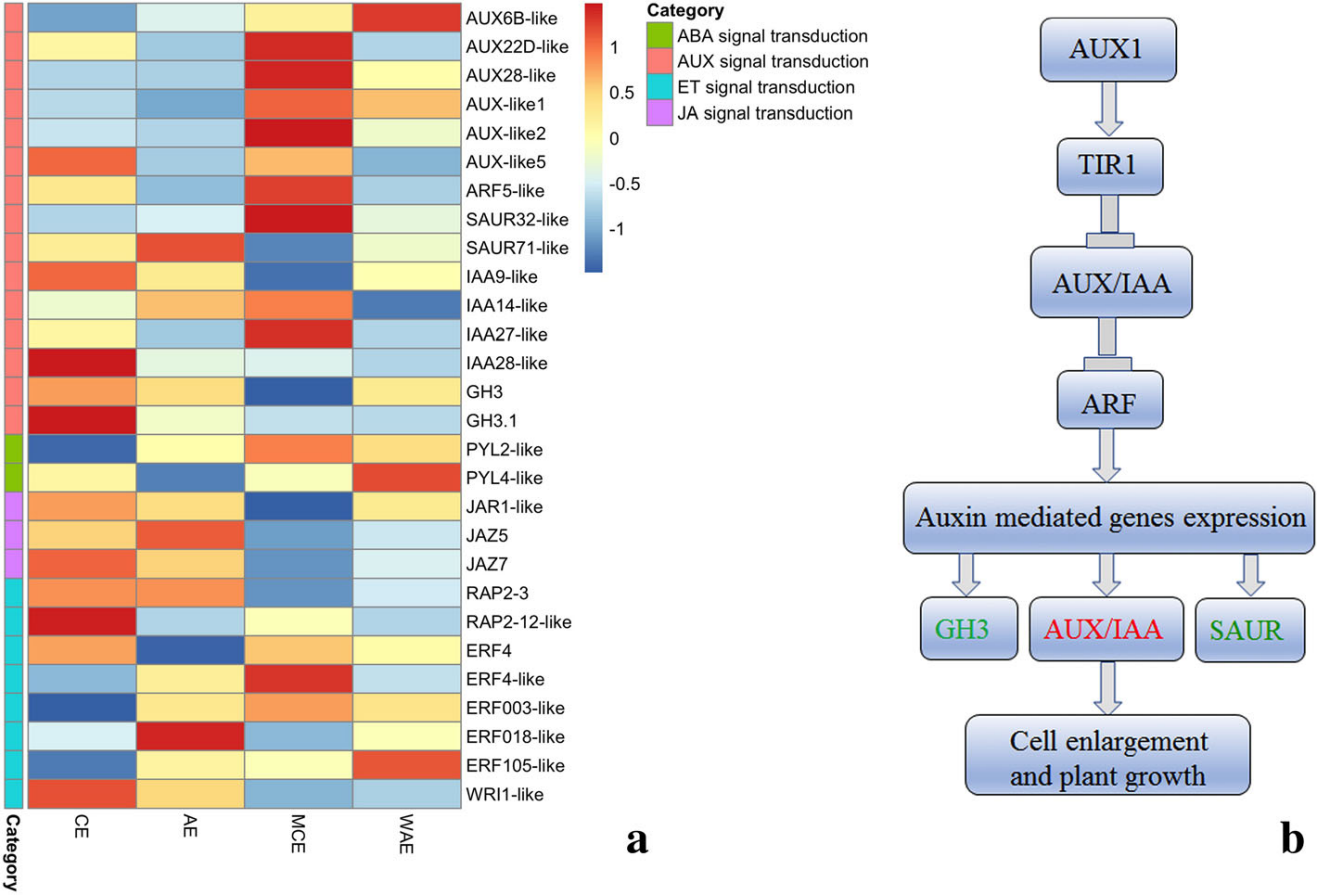
Heatmap of the differentially expressed genes in hormonal signaling transduction (**a**) and putative pathway for AUX signaling (**b**). Heatmap indicate the gene expression level by Log_2_ [FPKM] with a rainbow color scale. CE: cotyledonary embryo; AE: abnormal embryo; MCE: mature cotyledonary embryo; WAE: withered abnormal embryo

Various phytohormones induced SE and regeneration in several plants have already been reported. For instance, auxin was used alone or in combination with other plant growth regulators on plant SE induction [43, 44]. To further understand hormone regulation, FPKMs of hormonal signal transduction related genes were analyzed (Fig. 7a and Table S1). Among auxin signal transduction related genes, *AUX-like5*, *IAA9-like*, *IAA28-like* and *GH3.1* were up-regulated in CE. *SAUR71-like* were highly expressed in AE than in CE. *AUX22D-like*, *AUX28-like*, *AUX-like1*, *AUX-like2*, *SAUR32-like*, *IAA14-like* and *IAA27-like* were highly expressed in MCE. *ARF5-like* was lowly expressed in CE but highly expressed in MCE. These genes participated in the auxin signaling pathway, which was important for cell enlargement and plant growth (Fig. 7b).

Among abscisic acid (ABA) signal transduction related genes, *PYL2-like* was down-regulated in CE. *PYL4-like* was down-regulated in AE. Among jasmonic acid (JA) signal transduction related genes, *JAZ7* was highly expressed in CE than in AE. *JAZ5* was up-regulated in AE. Among ethylene (ET) signal transduction related genes, *RAP2–3* was up-regulated in CE and in AE. *RAP2–12-like* and *WRI1-like* were highly expressed in CE. *ERF4-like* was up-regulated in MCE. *ERF018-like* was only up-regulated in AE. All the genes involved in the hormones signaling transduction pathways, including auxin, ABA, JA, ET, suggested that these hormones had an indispensable role in their complicated crosstalk process during *H. brasiliensis* late SE. In vitro studies have suggested the role of various regulatory genes in embryogenic transition that are triggered by plant hormones [44]. The dynamic changes of these genes expression were critical for development of SEs.

### Differential expression of TFs and SE-related genes between CE and AE

**Fig. 8:**
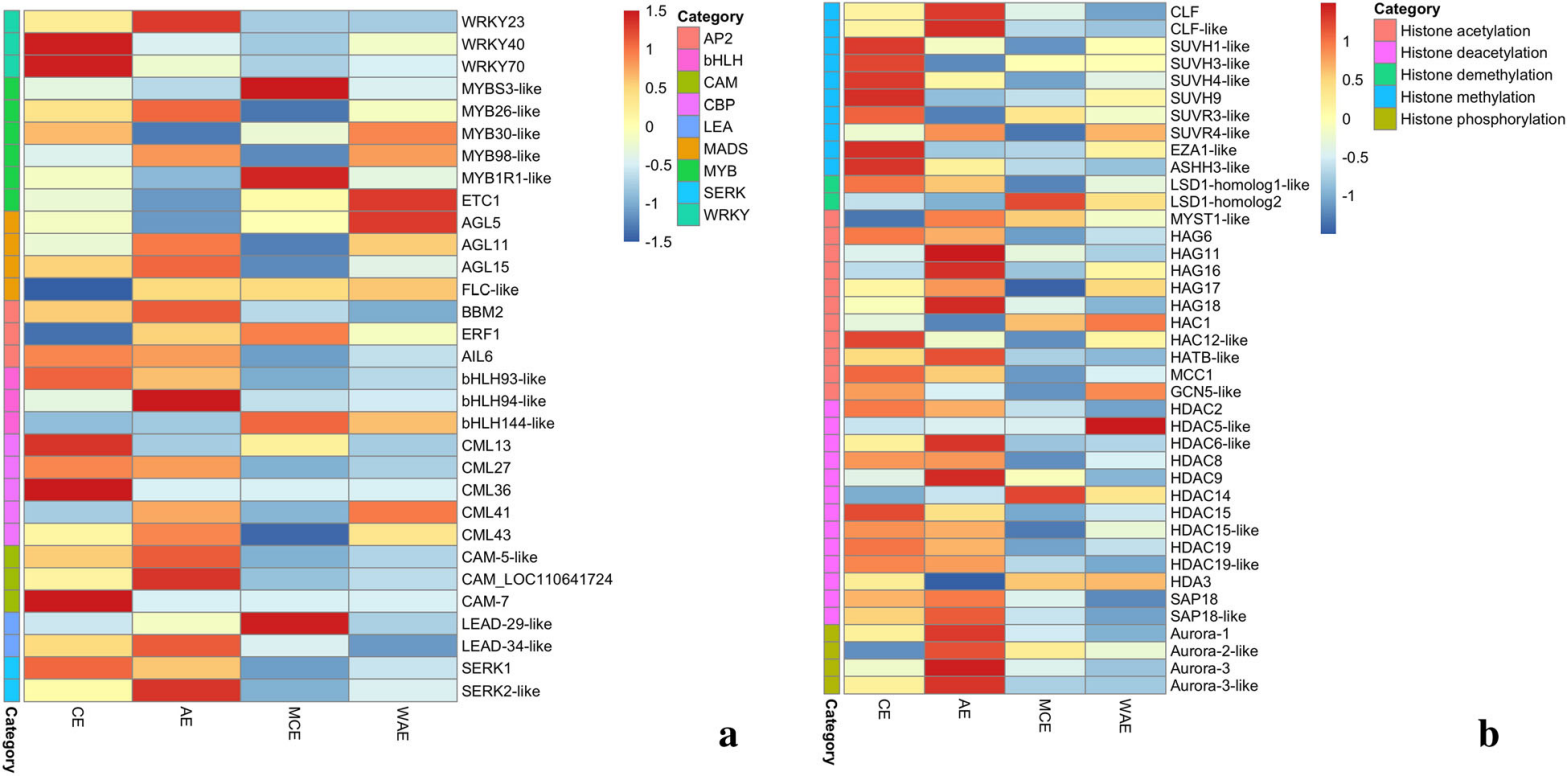
Analysis of the differentially expressed genes in CE, AE, MCE and WAE. **a** Heatmap of the differentially expressed TFs and SE-related genes. **b** Heatmap of the differentially expressed in histone modifications. Heatmap indicate the gene expression level by Log_2_ [FPKM] with a rainbow color scale. CE: cotyledonary embryo; AE: abnormal embryo; MCE: mature cotyledonary embryo; WAE: withered abnormal embryo

Transcription factors (TFs) play important roles in hormone signaling and stress responses as multifunctional regulators in both zygotic embryo and SE. Some of these TFs have been used as markers of totipotency in plant species [45]. In the present study, we show that several TFs might play an important role during late SE of *H. brasiliensis*. In this regard, 219 TFs were identified. The following TFs families were overrepresented: WRKY, MYB, MADS-box, AP2/ERF, bHLH. The expression profiles of 19 TFs in CE, AE, MCE and WAE are shown in Fig. 8a and Table S2. *WRKY40* and *WRKY70* were up-regulated in CE and down-regulated in AE. *WRKY23* were highly expressed in AE than in CE. *MYB26-like and MYB98-like* were up-regulated in AE. *MYBS3-like* and *MYB1R1*-*like* were up-regulated in MCE. *AGL11* and *AGL15* were up-regulated in AE. *BBM2* was highly expressed in AE. *AIL6* was highly expressed in CE than in AE. *bHLH93-like* was highly expressed in CE. The expression of *bHLH94-like* was up-regulated in AE. The results implied these TFs may play a key role in *H. brasiliensis* late SE.

Some SE-related genes, such as CAM [46], SERK [47, 48], LEA [49, 50], have been identified to play a vital role during plant embryogenesis. *CML13* and *CML36* were up-regulated in CE but down-regulated in AE. *CAM-5-like* and *CAM* (LOC110641724) were up-regulated in AE but had not changed in CE. *CAM-7* was up-regulated in CE but down-regulated in AE. *SERK1* was up-regulated in CE. *LEAD-34-like* and *SERK2-like* showed higher expression in AE than in CE. *LEAD-29-like* was up-regulated in MCE. The dynamic variation of the FPKM of these somatic embryogenesis-related genes suggested that they were critical for *H. brasiliensis* late SE.

### Differential expression of histone modifications related genes between CE and AE

The plant growth regulators and abiotic stress can contribute SE. In the meantime, these factors may induce epigenetic modifications [51]. Histone modification is one of the most important epigenetic modifications and plays a key role in the regulation of gene expression [52]. Therefore, the expression levels of histone modifiers were analyzed and shown in Fig. 8b and Table S3. *CURLY LEAF (CLF)*, encoding one of polycomb repressive complex 2 (PRC2) catalytic subunit that repress gene expression through trimethylating histone H3 at lysine 27 (H3K27me3), was higher expression in AE than in CE. The histone H3 lysine 9 methyltransferase genes (*SUVH1-like*, *SUVH3-like*, *SUVH4-like* and *SUVH9*), *SUVR3-like*, *EZA1-like* and *ASHH3-like* were expressed at a higher level in CE. In addition, histone demethylation related genes, *LSD1-homolog 1-like* was highly expressed in CE. *LSD1-homolog 2* was up-regulated in MCE. The increased expression of genes in CE or MCE suggested that it is likely to have a function during late SE.

The acetylation of histones is believed to promote open chromatin state and activate gene transcription. Ten of the eleven genes related to histone acetylation showed significant differential expression in CE vs. AE. *HAG6*, *HAC12-like*, *MCC1* and *GCN5-like* were up-regulated in CE. *HAG11*, *HAG16*, *HAG18* and *HATB-like* were up-regulated in AE. 7 of the 13 genes related to histone deacetylation showed an obvious difference of expression in CE vs. AE. *HDAC15-like* and *HDAC19* were highly expressed in CE. *HDAC6-like*, *HDAC9* and *SAP18-like* were up-regulated in AE.

The histone phosphorylation related genes (*Aurora-1*, *Aurora-2 like, Aurora-3* and *Aurora-3 like*) were highly expressed in AE than in CE. Plant Auroras can be divided into two categories according to the functions of Auroras. The alpha Auroras (Aurora 1 and Aurora 2) involve in controlling formative divisions throughout plant development. The beta Aurora (Aurora 3) associate with chromosome separation [53]. These genes highly expressed in AE can be used as candidate genes for in-depth study in vitro embryogenesis.

### qPCR verification of selected DEGs

**Fig. 9:**
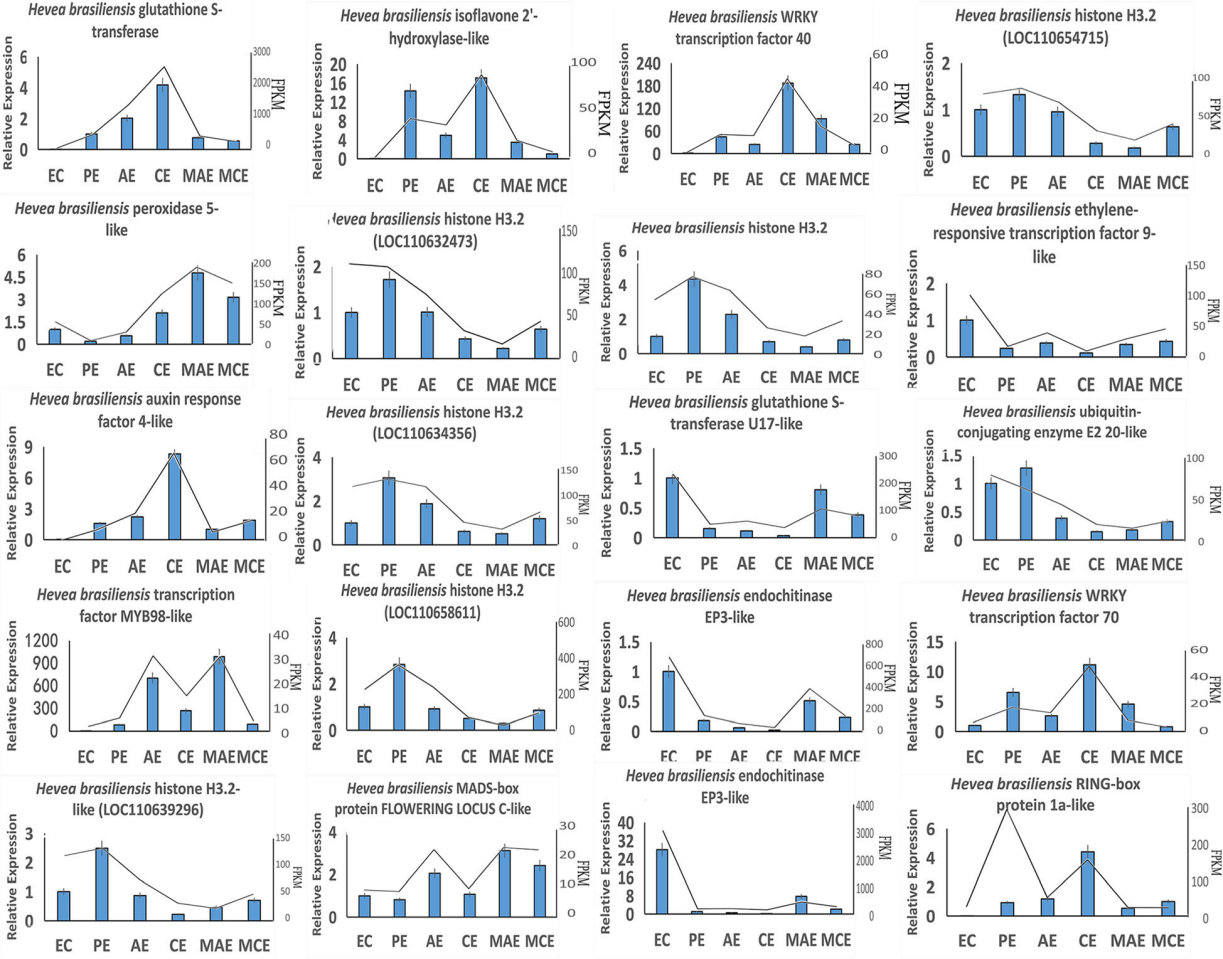
qRT-PCR verification of the selected DEGs involved in EC, PE, CE, AE, MCE, and WAE. The *H. brasiliensis* DEGs selected on the basis of their annotation. The data of polyline derived from FPKM of each gene. The 2^-ΔΔCt^ method was used to calculate the relative expression levels of genes. The statistical differences were analyzed by ANOVA (One-way analysis of variance) based on Fisher’s LSD (*P* < 0.05 and *P* < 0.01). EC: embryogenic callus; PE: primary embryo; CE: cotyledonary embryo; AE: abnormal embryo; MCE: mature cotyledonary embryo; WAE: withered abnormal embryo

To verify the reliability of transcriptome data, twenty genes related to SE were selected to carry out expression level analysis using qRT-PCR across 6 different tissues of *H. brasiliensis* (Fig. 9). Based on the transcriptome data analysis of *H. brasiliensis* SE, *ARF4-like, GST, I2’H-like*, *PRX5-like, RBX1a-like*, *WRKY40* and *WRKY70* were highly expressed in CE than in AE. *E2 20-like*, two *EP3-likes*, *ERF9-like*, *FLC-like*, five *H3.2* genes, *H3.2-like*, *MYB98-like* and U17-like were lowly expressed in CE than in AE. The qPCR results validated the expression levels of 19 genes which were highly consistent with transcriptome data.

## Discussion

SE is a promising and rapid vegetative propagation technique for plant regeneration. However, the process of SE remains poorly understood and many factors impact upon competence for SE. Many problems need to be resolved and one of these could be a deep understanding of the molecular mechanisms involved either negatively o positively in the generation of the somatic embryos. The transcriptome analysis of plant SE revealed a large number of potential key factors of embryogenesis [25, 26, 54–56]. In longan early SE, 27 SE specific or preferential genes and 28 NEC (Non-embryogenic callus) preferential genes were characterized as molecular markers genes for longan early SE. The NEC-specific marker genes maybe the key inhibitor of the transition from NEC to EC, while the SE markers may function on SE development [26]. In this study, we obtained the transcriptome analysis of rubber tree SE derived from EC, PE, CE, AE, MCE and WAE. The de novo assembly generated 36,937 unigenes. We found the regenerate competence of CE and AE had obvious differences during late SE. Therefore, this study mainly focused on DEGs in CE vs. AE and MCE vs. WAE.

In CE vs. AE, 376 DEGs were identified and assigned to 46 KEGG pathways. The 771 DEGs were also assigned to 57 KEGG pathways in MCE vs. WAE. The most representative pathways were phytohormones signaling pathway, biosynthesis of phenylpropanoid, and sucrose and starch metabolism in CE vs. AE and MCE vs. WAE. The significant role of phenylpropanoid biosynthesis in plant SE development has been studied, this pathway is associated with the tolerance of stress responses, probably through the reinforcement of the cell wall [57]. The phenylpropanoid biosynthesis-related genes were significantly enriched in papaya embryogenic callus [25] and in strawberry embryogenic callus [19]. In addition, external stimuli and plant hormones related genes played a key role in the SE process [58, 59]. In longan early SE, plant hormones related genes were enriched, especially the cytokinin and auxin signaling components [26]. Moreover, signaling involved in sucrose and starch accumulation is essential for somatic embryogenetic development [60]. The nature of the carbohydrate supply can reflect the signaling networks that control development, including somatic embryogenesis [61]. Sucrose was added to the culture medium as exogenous carbon sources in conifers SE [62, 63]. The germination of Norway spruce (*Picea abies*) somatic embryos was affected by carbohydrates [64]. Endogenous carbohydrate status varies throughout the somatic embryogenesis of conifers [65], and can be used to identify cell lines with high-quality embryos [66, 67]. Genes involved in the three pathways can play important role in *H. brasiliensis* late SE.

Auxin is critical regulator in different developmental stages of SEs [68–70]. The addition of exogenous auxin can affect the expression level of endogenous IAA [59, 71–73]. Dynamic change of endogenous IAA has been proved to induce plant SE and improve SE competency [74]. *Auxin/Indole-3-Acetic Acids (Aux/IAAs)*, *Gretchen Hagen3s (GH3s)*, *small auxin upregulated RNAs (SAURs)* and *auxin response factor (ARF)* have been identified as auxin-responsive genes in auxin signaling and homeostasis [75–77], which can regulate downstream genes precisely and rapidly, and further regulated plant growth and developmental processes. Aux/IAA family plays a key role in inhibiting the expression levels of genes transcriptional activated by ARFs [78, 79]. In low auxin levels, Aux/IAA proteins interacted with ARFs and inhibited activation of auxin-responsive genes. In high auxin levels, these proteins can interact with transport inhibitor response 1/auxin signaling F-box (TIR1/AFB) receptors to be ubiquitinated and subsequently resolved by the 26S proteasome [80–82]. The liberated ARFs regulated the expression of auxin-responsive genes (Fig. 7b). There were 29 Aux/IAA family members in *Arabidopsis*, but not all of them were induced by auxin [83]. Many Aux/IAA genes have also been identified in other plants, such as, *Eucalyptus grandis* [84]*, Solanum Lycopersicon* [85]*, Cucumis sativus* [86]*, Populus trichocarpa* [87]*, Zea mays* [88] and *Oryza sativa* [89, 90]. SAUR genes were consisted of a large multigene family, played crucial roles in regulating plant growth and development [91, 92]. GH3 family participated in a series of hormone-dependent processes in plant, including root growth, and flowering [93, 94]. In this study, high concentration of IAA and 2, 4-D were added in MS medium for inducing EC from immature male flowers. The concentration of IAA and 2, 4-D were reduced and withdrawal in the medium to trigger SE. This helps to slow down callus growth to induce embryo formation [95]. The transition was connected with changes in gene expression. Some *AUX*/*IAA* family genes were highly expressed in CE or MCE. *GH3.1* was up-regulated in CE. *SAUR32-like* and *ARF5-like* were up-regulated in MCE. These genes could be good gene expression markers and play a key role in the embryogenesis development process. In addition, JA and ET have also been reported to play a role in SE induction [96]. *JAZ7*, *RAP2–12-like* and *WRI1-like* were highly expressed in CE. The phytohormones signaling pathway related genes displayed intricate regulation during *H. brasiliensis* late SE. The regulatory mechanisms of these genes in *H. brasiliensis* late SE will be confirmed in the future study.

Transcription factors are key factors in plant embryogenesis and development. Many studies on SE development showed that complicated transcription regulation networks maintaining embryogenic competency, and embryogenic callus formation [63, 97]. Some members of the WRKY TFs family genes can be stimulated by stress and are involved in carpel and ovule as well as in embryogenesis development [98, 99]. Some WRKY genes have also been reported to be upregulated in embryogenic callus formation of bread wheat [54]. Transcriptome analysis showed that some WRKY genes are inducible in papaya and *Arabidopsis thaliana* embryogenic callus [25, 100]. In *Panax ginseng*, the expression of *PgWRKY6* increased in SE process in response to 2, 4-D inducing. PgWRKY6 functioned in the development of embryogenic callus possibly through the signaling cross-link of auxins with reactive oxygen species in somatic embryogenesis [101]. These finds indicates WRKY TFs have crucial role in the process of somatic embryogenesis. To our best knowledge, there is no report on WRKY TFs regulating genes associated with SE. MYB family was also involved in plant development and growth [102–105], hormone signal transduction [106, 107]. In this study, *WRKY40*, *WRKY70*, *MYBS3-like* and *MYB1R1*-*like* were highly expressed in CE, suggesting that they might be used as marker genes for *H. brasiliensis* late SE. *WRKY23*, *MYB26-like* and *MYB98-like* were up-regulated in AE, indicating that these genes might act as negative modulators of SE. In addition, AtEMK, a member of the AP2/ERF family, was ectopically expressed and promotes the initiation of somatic embryos in *Arabidopsis* and *H. brasiliensis* [14, 108]. BBM had been reported as a marker in *Brassica napus* SE [109]. The over-expression of BBM can enhance SE and regeneration ability in tobacco, sweet pepper, cacao [40, 110, 111]. The bHLH family is involved in developmental, growth, abiotic stress responses [112], and axillary meristem formation [63]. They also participate in abscisic acid and brassinosteroid signaling in *Arabidopsis* and rice [113]. A member of bHLH protein BIM1 regulated *Arabidopsis* SE and be involved in auxin and BR signaling pathways [114]. In this study, *AIL6* and *bHLH93-like* were highly expressed in CE, suggesting that they might play a key role in *H. brasiliensis* late SE. *AGL11*, *AGL15*, *BBM2* and *bHLH94-like* were up-regulated in AE, indicating that they have a negative regulatory role in late SE. To our knowledge, few transcription factors have been identified as negative modulators of plant SE. It will be of great interest to elucidate the function of these genes as negative modulators of SE. SERK has been proved as a key factor in plant SE. *AtSERK1* was higher expression during *Arabidopsis* embryogenic formation [115]. SERK was abundant in embryogenic tissues in *Dactylis glomerate* [116]. However, SERKs were also tested in non-embryogenic tissues in maize, rice and wheat [47, 117, 118]. Ca^2+^ has been identified to play a mediating role during plant SE [46, 119]. *LEA5*, a late embryogenesis abundant proteins gene, was highly expressed in late embryogenesis [120]. In this study, *SERK1, CML13, CML36* and *CAM-7* were up-regulated in CE. *LEAD-29-like* were up-regulated in MCE. These genes can have various regulatory functions in *H. brasiliensis* late SE. *LEAD-34-like* and *SERK2-like* werehighly expressed in AE than CE, implying that they acted as negative modulators of SE. Further investigation of regulatory machinery of these genes will be important in improving natural rubber SE.

The histone modifications played important roles in gene expression, DNA replication and transcription, chromatin compaction [121, 122]. Histone lysine methylations possessed the function of activating or derepressing transcription. H3K4, H3K36 and H3K79 methylations are associated with active transcription, whereas, H3K9, H3K27 and H4K20 methylations are involved in gene silencing [123]. H3K27me3 and H3K4me3 are the most frequent histone methylation marks. H3K27me3 is catalyzed by the trithorax-group (TrxG) and polycomb-group (PcG) proteins, of documented roles in regulating plant responses to environmental cues, cellular reprogramming, and plant stem cell maintenance [124]. The PcG proteins (PRC1 and PRC2), which cooperate to repress the genes via histone methylation during plant development [125]. In this study, *CLF* was higher expression in AE, suggesting H3K27me3 might inhibit the expression of genes associated with SE. Seven histone methylation related genes (*SUVH1-like*, *SUVH3-like*, *SUVH4-like*, *SUVH9*, *SUVR3-like*, *EZA1-like* and *ASHH3-like*) were expressed at a higher level in CE. In addition, histone demethylation related genes, *LSD1-homolog 1-like* were highly expressed in CE. *LSD1-homolog 2* were only up-regulated in MCE. *KRYTONITE (KYP)*, encoding a histone H3 lysine 9 methyltransferase, also showed a higher expression level in *Arabidopsis* somatic embryos [16]. Some HATs including HAG1, HAF2, HAC1, HAC2, HAC4, HAC5 and HAC12 have been identified in *Arabidopsis* [16, 126, 127]. *HAC2, HAG2* and *HAG3* showed more accumulation in somatic embryos as compared to leaf tissues [16]. Similarly, in this study, histone acetylation related genes (*HAG6*, *HAC12-like*, *MCC1* and *GCN5-like*) and histone deacetylation related genes (*HDAC15-like*, *HDAC19*) showed higher expression in CE. *HDAC6-like*, *HDAC9* and *SAP18-like* were highly expressed in AE. HAC1 have been identified its function in reproductive and vegetative development [127]. HbHDA3 have been identified to interact with HbWRKY14 to regulate the expression of *HbSRPP* [128]. It is possible that those histone modifications related genes may also have an important function in embryogenesis. However, detection of changed transcript levels for key genes involved in histone modification provides an indirect indication of changed histone modifications during SE. It is not clear whether the expression changes we observed are due to in vitro conditions (i.e. externally supplied auxin, stress responses) or changed histone modification signatures. Therefore, it will be of great interest to perform a global analysis of the epigenome architecture of somatic embryos in order to underlying the relationship of the expression of genes associated with SE and histone modification.

## Conclusions

In this study, the transcriptome data for rubber tree SE were generated. A comparative analysis of gene expression profiles during rubber tree late SE identified a series of DEGs that regulated late SE in rubber tree. We revealed the expression level of some genes related to phytohormones signaling pathway such as auxin, JA and ET signaling pathway, implying their important roles in rubber tree embryogenesis development process. The transcription factors such as WRKY, MYB, AP2 and bHLH, as well as CAM, SERK and LEA that were related to rubber tree late SE, might play a key role and become potential molecular marker genes in late SE. Histone modification might have crucial roles during late SE. This study provides novel insights into the molecular regulation mechanisms during rubber tree late SE.

## Methods

### Plant material and induction of somatic embryogenesis

Plants of *Hevea brasiliensis* Muell. Arg. cultivar (reyan 7–33-97) were planted in National Rubber Tree Varieties Resource Garden of the Chinese Academy of Tropical Agriculture Sciences, Danzhou, Hainan, China.

Immature male flowers were gathered from the rubber tree of reyan7–33-37. Immature male flowers were surface-sterilized with 75% (v/v) ethanol for 30 s, and followed to immerse in 0.2% (v/v) mercuric chloride solution for 10 min, and then washed four times with distilled water. The immature anthers were cultured in solid MS medium containing 1 mg l^− 1^ 2,4-D, 1 mg l^− 1^ KT and 0.5 mg l^− 1^ NAA. After an additional 5–6 weeks of growth, EC were obtained in the darkness and 26–28 °C. These samples of PE, CE, AE, MCE and WAE were collected successively. All samples were rapidly frozen in liquid nitrogen, and stored at − 80 °C until RNA extraction. Three biological replicates were prepared for each sample.

### Construction of cDNA library and sequencing

Total RNA was extracted according to the instructions of RNAprep pure plant Kit (Polysaccharides and Poly phenolics-rich, QIAGEN). RNA degradation and contamination were monitored on 1% agarose gels. The quality of RNA was detected by using the NanoDrop 2000 spectrophotometer (IMPLEN, CA, USA). The mRNA was enriched from total RNA using magnetic beads containing Oligo (dT) and broken into small fragments. Transcriptome libraries were constructed according to the instructions of the Truseq™ RNA sample preparation kit from Illumina (San Diego, CA). The library quality was examined using the Qsep100 Analyzer (BIOptic Inc., Taiwan, China). The cDNA libraries were deep sequenced on the Illumina novaseq6000 cDNA sequencing platform.

### Transcriptome de novo assembly and annotation

RNA seq data were quality controlled using SeqPrep (https://github.com/jstjohn/Seq-Prep) and Sickle (https://github.com/najoshi/sickle) with default parameters. Clean reads were acquired to remove the reads with adaptor sequences and ambiguous “N” base more than 1% and base quality less than Q15. All clean data were employed to do de novo assembly using Trinity (http://trinityrnaseq.sourceforge.net/). All unigenes were identified by searching the *H. brasiliensis* genome (GenBank under the accession code of LVXX01000000) from NCBI (https://www.ncbi.nlm.nih.gov/). All unigenes were searched against the COG (http://www.ncbi.nlm.nih.gov/COG), GO (http://geneontology.org/), KOG (http://www.ncbi.nlm.nih.gov/structure/cdd/cdd.shtm) and eggNOG (http://eggnog5.embl.de/#/app/home) databases using BLASTX (E-value–5 ≤ 10). BLAST2GO program (http://www.blast2go.com/b2ghome) was employed to get GO annotations of unique assembled unigenes for describing BP, MF and CC. The KEGG (https://www.kegg.jp) was to analyze metabolic pathway.

### Analysis of differentially expressed genes (DEGs)

The expression level of the unigenes was calculated by FPKM. The FC represented the ratio of FPKM between two samples. The Benjamini-Hochberg correction method was adopted to correct the significance *P*-value obtained from the original hypothesis test. FDR was obtained by correcting the P-value of different significance. The genes with − 1.0 ≥ Log_2_ [FC] ≥ 1.0 and the threshold of FDR < 0.01 were regarded as DEGs. A Venn diagram was created to find the overlapped DEGs in different developmental stages of *H. brasiliensis* SE using VennMaster as described previously [129].

### Expression profiles of genes in *H. brasiliensis* SE

FPKM was applied to analyze the gene expression level. The heat map was created using log_2_ [FPKM] with the pheatmap package [130].

### Quantitative PCR (qPCR)

Twenty genes were chosen for validation by qPCR. The samples of EC, PE, CE, AE, MCE and WAE were used for RNA extraction, and then reverse transcribed into cDNA as template. Each sample included three biological replicates. qPCR specific primers for the twenty genes were designed by using Primer Premier software 6.0 (Table S4). HbACT7 was amplified as a standard control as described previously [131]. qPCR was performed on a Mx3005P Real-Time PCR system using a SYBR Premix EXTaq II™ Kit (TaKaRa, China). All reactions were performed at 95 °C for 30 s, 40 cycles at 95 °C for 10 s, 58 °C for 20 s, and 72 °C for 25 s. The 2^-ΔΔCt^ method was used to calculate the relative expression levels of genes [132]. The statistical differences were analyzed by ANOVA (One-way analysis of variance) based on Fisher’s LSD test (*P* < 0.05 and *P* < 0.01) [133].

## Supplementary Information

**Additional file 1: Figure S1.** GO function of classification of consensus.

**Additional file 2: Figure S2.** KOG function of classification of consensus.

**Additional file 3: Figure S3.** eggNOG function of classification of consensus.

**Additional file 4: Table S1.** FPKM and annotation of hormonal signal transduction related genes.

**Additional file 5: Table S2.** FPKM and annotation of TFs and SE-related genes.

**Additional file 6: Table S3.** FPKM and annotation of histone modifications related genes.

**Additional file 7: Table S4** qRT-PCR Primer.

## Data Availability

The generated RNA-seq data have been deposited in NCBI-SRA database under the accession of PRJNA646309. The *H. brasiliensis* genome data used to annotate unigenes was downloaded from NCBI (GenBank under the accession codes of LVXX01000000). The annotation of the top 20 expressed genes in EC, PE, CE, and MCE library, histone modifications related genes, hormonal signal transduction related genes, TFs, and SE-related genes were deposited in the NCBI database. The accession numbers are included in Table 3 and Tables S1, S2, S3.

## Abbreviations

SE: Somatic embryogenesis
EC: Embryogenic callus
PE: Primary embryo
CE: Cotyledonary embryo
AE: Abnormal embryo
MCE: Mature cotyledonary embryo
WAE: Withered abnormal embryo
DEGs: Differentially expressed genes
SRJCs: Self-rooted juvenile clones
LEA: Late embryogenesis abundant protein
SERK: Somatic embryogenesis receptor-like kinase
BBM: BABY BOOM
MS: Murashige and Skoog
2,4-D: 2,4-Dichlorophenoxyacetic acid
KT: Kinetin
NAA: Naphthylacetic acid
IAA: Indole-3-acetic acid
GA_3_: Gibberellic acid
6-BA: 6-benzyl aminopurine
COG: Clusters of orthologous groups of proteins database
GO: Gene ontology
KOG: EuKaryotic orthologous groups
egg NOG: Non-supervised orthologous groups
KEGG: Kyoto encyclopedia of genes and genomes
FPKM: Fragments per kilo base of transcript sequence per million base pairs sequenced
*GST*: *Glutathione S-transferase*
*LTP*: *Lipid-transfer protein*
*POD*: *Peroxidase*
FC: Fold change
FDR: False discovery rate
BP: Biological process
CC: Cellular component
MF: Molecular function
cDNA: Complementary DNA
ABA: Abscisic acid
JA: Jasmonic acid
ET: Ethylene
TFs: Transcription factors
HATs: Histone acetyltransferases
qPCR: Quantitative polymerase chain reaction
*Aux/IAAs*: *Auxin/indole-3-acetic acids*
*GH3s*: *Gretchen hagen3s*
*SAURs*: *Small auxin upregulated RNAs*
*ARF*: *Auxin response factor*
TIR1/AFB: Transport inhibitor response 1/ auxin signaling F-box
KYP: KRYTONITE
ANOVA: One-way analysis of variance
NEC: Non-embryogenic callus
ICpEC: Incomplete compact pro-embryogenic cultures
GE: Globular embryos
