# Supplementary figures and images for: Transcriptomes analysis reveals novel insight into the molecular mechanisms of somatic embryogenesis in Hevea brasiliensis
Source: BMC Genomics. 2021 Mar 12;22:183. doi: 10.1186/s12864-021-07501-9 (PMC7953812; doi:10.1186/s12864-021-07501-9)

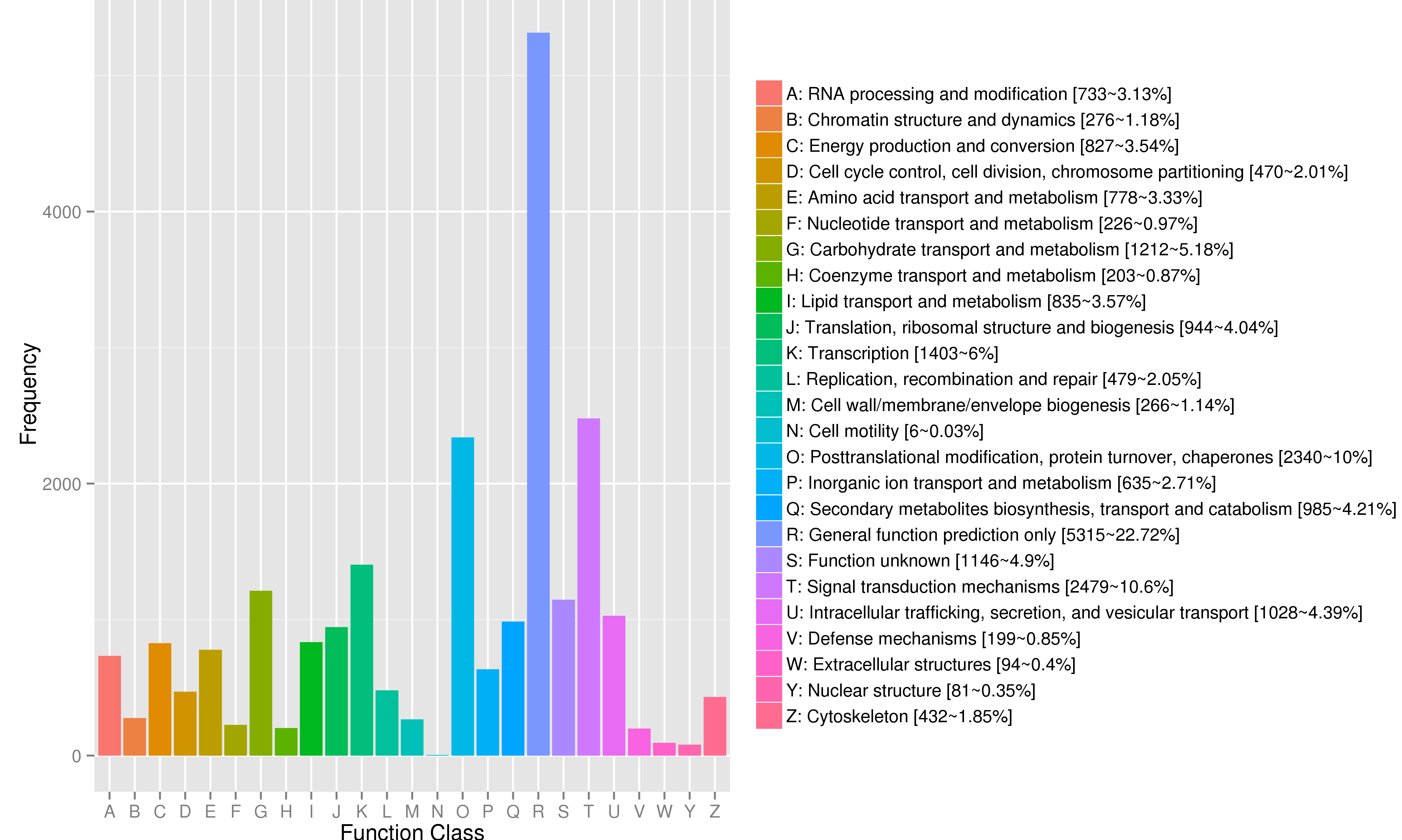

Supplement: Supplementary file 1 — Additional file 1: Figure S1. GO function of classification of consensus. [file 12864_2021_7501_MOESM1_ESM.tif]

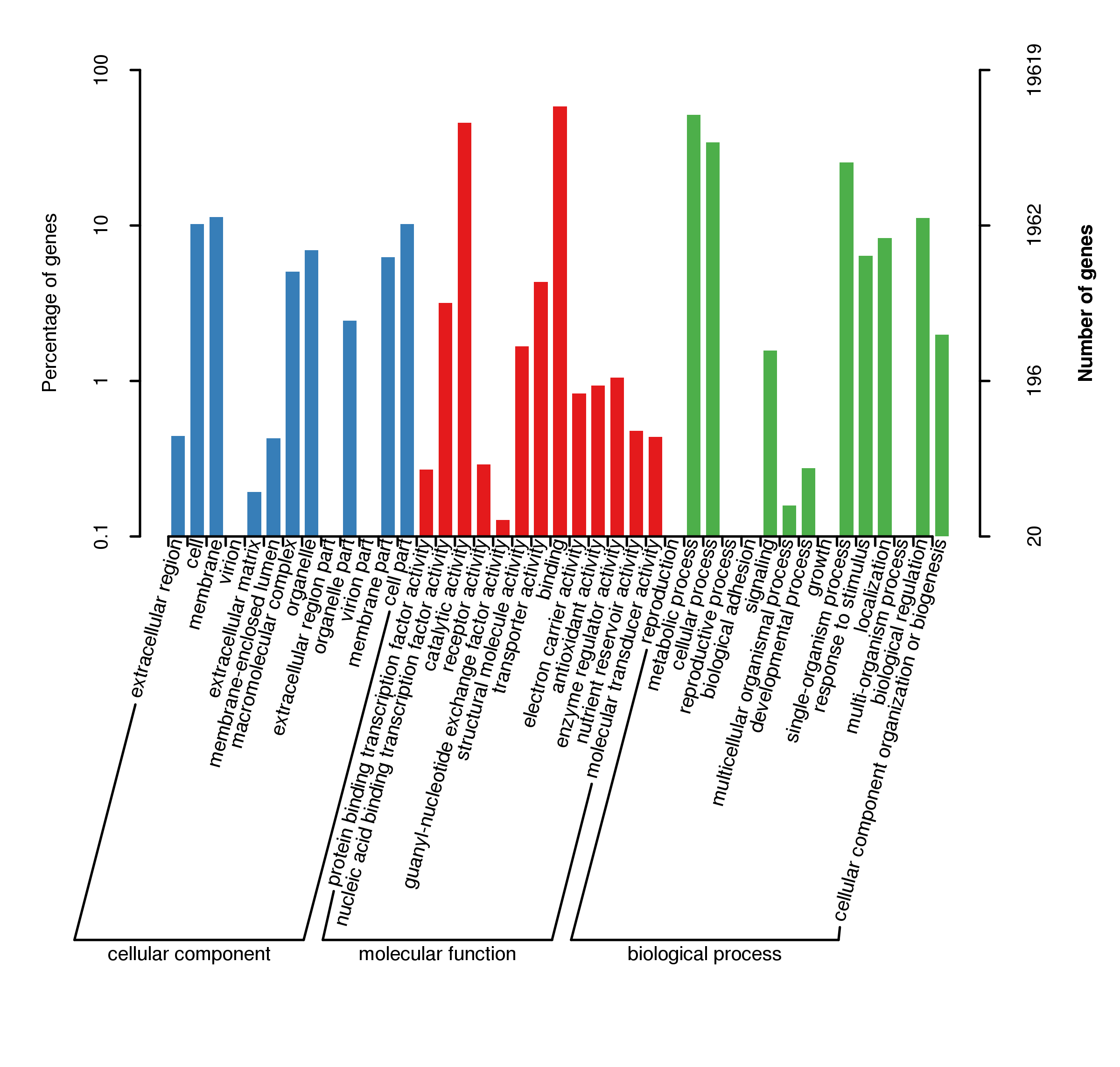

Supplement: Supplementary file 2 — Additional file 2: Figure S2. KOG function of classification of consensus. [file 12864_2021_7501_MOESM2_ESM.tif]

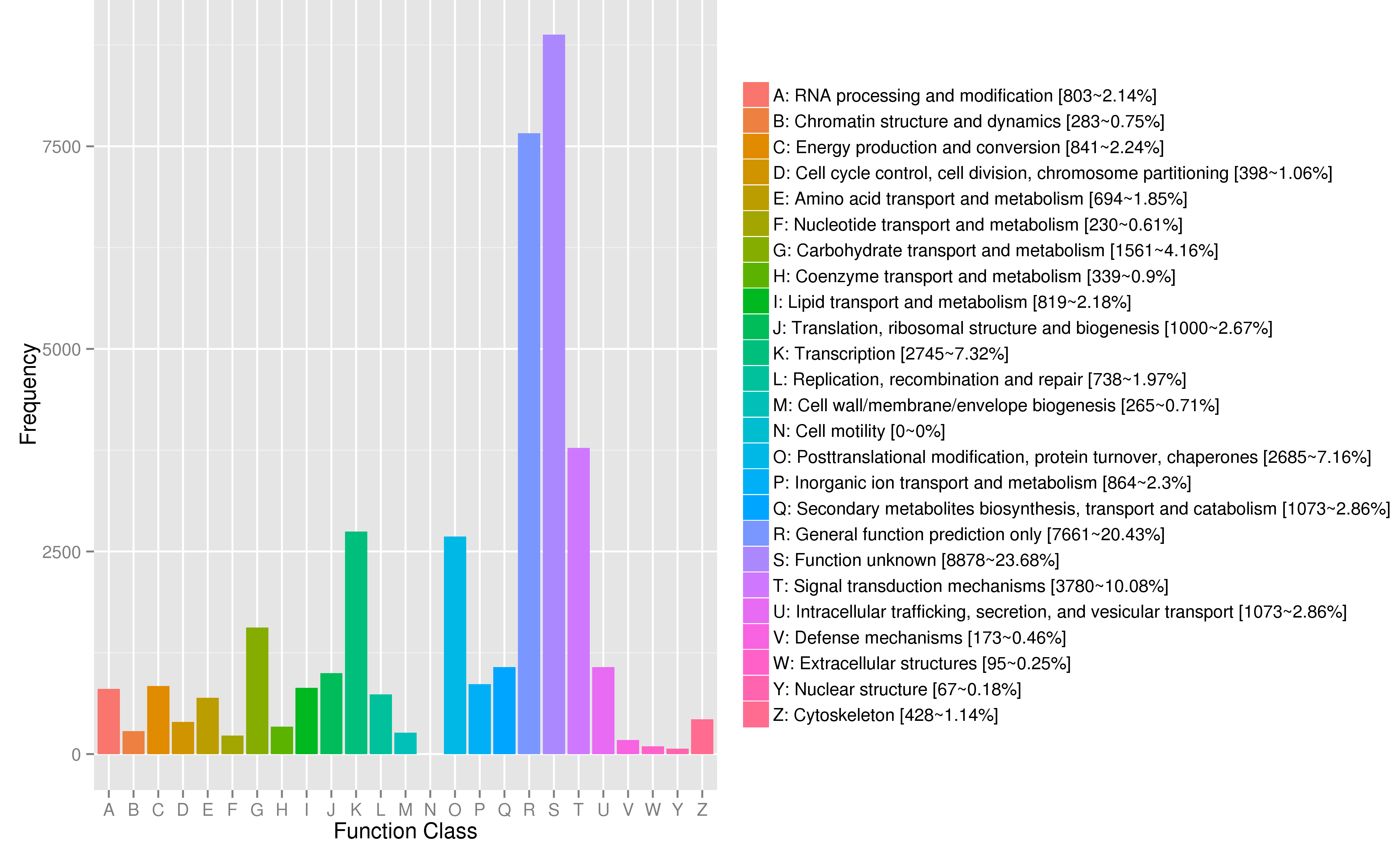

Supplement: Supplementary file 3 — Additional file 3: Figure S3. eggNOG function of classification of consensus. [file 12864_2021_7501_MOESM3_ESM.tif]
