# Supplementary material for: Transcriptomes analysis reveals novel insight into the molecular mechanisms of somatic embryogenesis in Hevea brasiliensis
Source: BMC Genomics. 2021 Mar 12;22:183. doi: 10.1186/s12864-021-07501-9 (PMC7953812; doi:10.1186/s12864-021-07501-9)
Supplement: Supplementary file 7 — Additional file 7: Table S4 qRT-PCR Primer. [file 12864_2021_7501_MOESM7_ESM.docx]

**Table S4 qRT-PCR Primer**

| Gene | Forward primer（5’-3’） | Reverse primer（5’-3’） |
| --- | --- | --- |
| auxin response factor 4-like | GGAGATTATCCATGGCAGGAA | GTTTCCCATACATAAACGCATCTC |
| isoflavone 2'-hydroxylase-like | GGGATAAGGTTTACTGTACCCAA | CCTGGAAGATCACATAAGTGTAGAA |
| peroxidase 5-like | TGCATGGGCTTCCAAGTT | TGCTCATAAACAAATGGCCAAG |
| RING-box protein 1a-like | TGTGCAATTTGTAGGAACCATA | TCTAATGTCCGTACTTCTGGAAT |
| WRKY transcription factor 40 | TGCAACTCTAAGGTTGAAGAGTA | GTACAGTAGAAGCATAAGCAATCAA |
| WRKY transcription factor 70 | CTCACCTACAACCAATCATCTTCT | GCCATCGAAGCATTCAAATTCC |
| glutathione S-transferase | TTCCAAATTTATCTGGATGGATGC | GGAGCAGAGATAACAATTAGAAGAAAG |
| transcription factor MYB98-like | TTGTGTGGACGCAAACTTATTG | TGGGTGATCATCTCTAGCAAATC |
| histone H3.2 | CTCTGTGCTATTCACGCTAAGA | CCATCACGACTGCAAATTGAT |
| histone H3.2 (LOC110632473) | ATTCACGCTAAGAGGGTTACTATC | TCCATAGGATTTCTTCCTCAACTAC |
| histone H3.2 (LOC110634356) | TTCAAGACAGATCTGAGGTTTCA | AGGACCATTGAAATCGACTCTT |
| histone H3.2-like | TATCCAGCTAGCAAGGAGGATTAG | TCGTTTACTGCTTAGGAGGCA |
| histone H3.2 (LOC110654715) | GCTATTCATGCTAAGAGGGTTACT | GACGAGACTTTCCACTGGATTT |
| histone H3.2 (LOC110658611) | CTGTGCTATTCACGCTAAGAGA | GCTTAGCCAATACAAAGATAAGGAC |
| ethylene-responsive transcription factor 9-like | GGACTCATCATCTGTGGTTGAC | ACATCCAATGATAACCCAGTAACTA |
| Glutathione-S-transferase U17-like | AAATGGGCTGACACCTTCTC | AAACAACACACAAGGCATACAC |
| ubiquitin-conjugating enzyme E2 20-like | GCTCGCCTCTAAACACTCAA | AAAGCTTCGTTCCACCCTTC |
| endochitinase EP3-like | ACCTGCCAAAGTTAAAGCTAGA | TCAAACTCGACCCACTCATAAA |
| MADS-box protein | GAGGGAGATGGAAGCAATGAA | GGAATCAGTGGACACATCACA |
| endochitinase EP3-like | ACCTGCCAAAGTTCAAGCTA | TCAAACTCGACCTTCTCCTAGA |
